# The Proper Business of the Coroner

**Published:** 1895-12-21

**Authors:** 


### The Hospital

AN INSTITUTIONAL JOURNAL OF

The Medical Sciences and Hospital Administration,

WITH

AN EXTRA NURSING SUPPLEMENT.

VOL. XIX.—No. 482 ] [DEC. 21, 1895.

### THE PROPER BUSINESS OF THE CORONER.

IT is often asked, who should be coroner; it is never asked who should be Archbishop of Canterbury or Lord Chief Justice of England. If any man were to propose a lawyer for the see of Canterbury or a clergyman for the Lord Chief Justiceship, he would very properly be considered a flippant person. Yet the business of the coroner is from first to last a medical business, as much as that of the Lord Chief Justice is judicial or that of the Archbishop of Canterbury is ecclesiastical and theological. What is the business of the coroner? It is simply and solely "to find out the cause of death." Is that the business of a medical man or of a lawyer? Merely to ask the question is to answer it, and in one only possible way.

Nobody who really takes the trouble to understand the question will deny that the proper person, and the only competent person, to investigate causes of death is the trained medical man; and so long as there is an adequate supply of trained medical men no responsible person in our times would think of suggesting any other than a medical man for the office of coroner if it were not that the office has been so often filled by solicitors and men of no particular training of any kind. This is just where the whole difficulty lies. The coroner's office has in the past been very largely filled by lawyers; and the average Englishman, endowed with but a dullish wit and an irresistible determination to stand by the methods of his grandfather, sees no earthly reason why the lawyer should not be coroner still, to the very end of time. Add to this the fact that the lawyer "in a lump" as the "Northern Farmer" put it, has an exceedingly keen scent for "plums," even if they be only damsons, and we see at once why this anomaly of setting a colonel to navigate a ship or a jack-tar to occupy a pulpit still flourishes in the midst of our scientific and growingly-reasonable times.

But, says the average man, must not a coroner be a person who is capable of understanding and putting a proper value upon "evidence"? Most true; and for that very reason the coroner must be a trained doctor and nothing else. A diamond dealer must be a man capable of understanding and valuing evidence, that is the evidence of the genuineness and value of diamonds he may be called upon to buy. And this illustration exposes at once the fallacy upon which the lawyer bases his claim to the coronership. The fallacy lies in the word "evidence." "Evidence" seems at once to connote lawyers. But is there no evidence except legal evidence? Did not the learned Paley publish a book on the "Evidences of Christianity"? Was that a kind of "evidence" which the lawyer alone could deal with? Paley's evidence was addressed, not to the specialist of any kind, but to the common man. The common man is therefore shown to be a man quite competent to deal with "evidence," provided always that the evidence be of a kind suited to his capacity, and of sufficient importance to be of interest to his mind.

Evidence then consists not merely of legal evidence, but of all kinds of evidence. The lawyer is naturally the most competent of men to deal with legal evidence, the clergyman with theological evidence, the sanitarian with sanitary evidence, the soldier with military evidence, and the doctor with evidence which concerns disease and death. Can anything be plainer? Would anybody think of making a lawyer a sanitary inspector? And yet it would be a hundred times easier to teach a lawyer to understand sanitary evidence than to teach him to understand the evidence relating to disease and death. The plain truth, and the whole truth, is this, that there is one and only one justification for the appointment of a lawyer as coroner, and that justification is the justification of precedent alone. In reason, in science, in the practical business of the question there is no more justification for a legal coroner than for a legal Archbishop of Canterbury or a legal physician to the Queen. And all this the lawyer knows as well as we do.

We have endeavoured in this article to limit attention exclusively to two points, because there are, in fact, only two points at issue. The first is the question of the true business of the coroner; and that, we have insisted, is "to find out the cause of death," and nothing else. In other words, the coroner's court is not a law court, but a medical, a "court of first instance," and that only. The second point is the claim of the lawyer to the coronership. That, we have shown, rests upon precedent, and upon precedent alone. It was established in days when scientific medicine and scientific methods of ascertaining causes of death were all but unknown. Since medicine, as to its methods and diagnostic results, has become absolutely scientific, the claim of the lawyer to hold investigations into causes of death is neither more nor less than an unscientific and absurd anachronism. The whole subject in its details is very fully discussed in a pamphlet by Mr. F. J. Lowndes, M.R.C.S., chief surgeon to the Liverpool police. To that pamphlet those who are interested are commended. The question is well worthy of study, and the final issue is in no manner of doubt. That the lawyer will ultimately be evicted everywhere from the coroner's office, for which, as we have shown, he has absolutely no specialised competence, is as certain as the progress of a scientific civilisation can make it.

